# Introduction

**DOI:** 10.1093/jbcr/irag033.000

**Published:** 2026-04-06

**Welcome to the ABA 2026 Annual Meeting Abstract Book**

On behalf of the ABA Annual Meeting Program Committee, welcome to the 2026 Abstract Book. This digital publication brings together the scientific and clinical contributions that were shared throughout this year’s meeting, reflecting the depth, innovation, and collaboration that continue to advance burn care and research.

Within these pages, readers will find peer-reviewed abstracts representing a wide range of disciplines, practice settings, and research interests. From emerging science and quality improvement initiatives to clinical outcomes and educational innovations, this collection highlights the work shaping the future of burn care.

This abstract book is designed to support meeting participation, session planning, and post-meeting reference. Whether preparing to attend a specific presentation, exploring new topic areas, or identifying collaborators, this resource is intended to serve as a practical and enduring reference throughout and beyond the 2026 Annual Meeting.

Thank you to the authors, reviewers, and committee members whose efforts made this program possible. The Program Committee looks forward to another engaging and impactful meeting.

**Angela Gibson, MD, PhD, FACS, FABA**

ABA Annual Meeting Program Committee Chair

**Amanda Bettencourt, RN, PhD, CCRN-K, ACCNS-P, APRN**

ABA Annual Meeting Program Committee Associate Chair

**Mandy Yelvington, PhD, OTR/L, BCPR, BT-C**

ABA Annual Meeting Program Committee Associate Chair

**How to Use the ABA 2026 Abstract Book**

This digital abstract book contains more than 600 abstracts organized by presentation format and topic area. The sections below outline how content is structured to help readers quickly locate material of interest.

**Abstract Presentation Categories**

Abstracts are grouped into the following primary sections:

- Aftercare and Reintegration
- Basic or Translational Sciences
- Burn and Wound Closure – Acute Surgical and Medical
- Critical Care and Medical Care
- Disaster/Mass Casualty/Firefighter Issue
- Diversity, Equity and Inclusion
- Epidemiology
- Ethics
- Fiscal, Economics and Leadership
- Nursing
- Pediatric
- Pharmacy
- Psychological and Psychosocial
- Public Health and Prevention
- Quality Improvement
- Reconstruction and Clinical Management of Scars
- Rehabilitation

**Top 5 Abstracts**

The Top 5 Abstracts include podium presentations selected for scientific excellence. These abstracts represent the highest-scoring submissions selected by the Program Committee and reviewers. They highlight exceptional scientific merit, innovation, and relevance to the burn care community.

**Correlative Sessions**

Correlative sessions group multiple related abstracts under a shared theme or topic area. Each correlative session includes several individual abstracts presented together in a structured format. In addition, the ABA accepted four Late Breaking Abstracts that were chosen for their high-impact, newly completed research.

**Quick Shots**

Quick Shot presentations feature concise research abstracts designed for rapid knowledge sharing and focused discussion.

*Traditional Posters*

Poster abstracts are traditional physical poster presentations organized by topic area and include a wide range of clinical, research, educational, and quality-improvement projects.

*ePosters*

ePosters are case study abstracts presented in digital format and organized by categories for easier navigation and browsing.

**Finding Specific Abstracts**

Because this is a digital publication, readers are encouraged to use the PDF search function to locate:

- Abstract numbers
- Author names
- Institutions
- Keywords or clinical topics
- Presentation titles

**Understanding Abstract Numbering**

Each abstract is assigned a presentation number for identification and reference.

Podium Abstracts are grouped under the Correlative sessions where they were presented, and numbered alphabetically by abstract title within that Correlative – e.g., C-752-03; C-855-07, etc.

Poster abstracts are sequentially numbered by presentation date and poster type.

Please refer to the Content Navigation below for more information.

**Content Navigation**

**Top 5 Abstracts:** Code: T-1 - T-5

**Correlative Sessions:** Code C-751 – C 953

- **C-751 – Correlative I:** Aftercare, Reintegration & Reconstruction
- **C-752 – Correlative II:** Burn and Wound Closure 1
- **C-753 – Correlative III:** Critical Care and Medical Care 1
- **C-754 – Correlative IV:** Basic or Translational Sciences & Pharmacy
- **C-755 – Correlative V:** Nursing & Epidemiology
- **C-756 – Correlative VI:** Pediatrics
- **C-851 – Correlative VII:** Burn and Wound Closure 2
- **C-852 – Correlative VIII:** Critical Care and Medical Care 2
- **C-853 – Correlative IX:** Rehabilitation, Quality Improvement, Pharmacy & Burn and Wound Care
- **C-854 – Correlative X:** Nursing
- **C-855 – Correlative XI:** Public Health and Prevention
- **C-856 – Correlative XII:** Psychological and Psychosocial
- **C-857 – Correlative XIII:** Ethics
- **C-951 – Correlative XIV:** Critical Care and Medical Care
- **C-952 – Correlative XV:** Quality Improvement
- **C-953 – Correlative XVI:** Fiscal, Economics and Leadership

**Quick Shots:** Code: C-954 – C-955

**Late Breaking Abstracts:** Code: C-955

Although Late Breaking Abstracts represent high-profile and timely research, they are placed at the end of the Abstract Book due to publishing timelines and production requirements.

**Posters**

**Traditional Posters:** 501-680 and 801-978

**ePosters (Case Studies):** 1001-1030 and 1101-1127

- **Aftercare and Reintegration:** 501-509
- **Basic or Translational Sciences:** 510-519, 801-810
- **Burn and Wound Closure:** 1001-1015, 1101-1112, 520-552, 811-844
- **Critical Care and Medical Care:** 1016-1018, 1113-1118, 553-574, 845-867
- **Disaster/Mass Casualty/Firefighter Issues:** 1019, 575-580
- **Diversity, Equity and Inclusion:** 868-875
- **Epidemiology:** 581-588, 876-883
- **Ethics:** 884-887
- **Fiscal, Economics and Leadership:** 888-894
- **Nursing:** 1020-1021, 589-596, 895-903
- **Pediatrics:** 1119, 597-603, 904-910
- **Pharmacy:** 604-610
- **Psychological and Psychosocial:** 1022, 1120-1121, 611-622, 911-923
- **Public Health and Prevention:** 623-637, 924-931
- **Quality Improvement:** 1023-1024, 1122, 638-661, 932-956
- **Reconstruction and Clinical Management of Scars:** 1025-1026, 1123-1124, 662-668, 957-964
- **Rehabilitation:** 1027-1030, 1125-1127, 669-680, 965-978

**Using This Resource After the Meeting**

In addition to supporting on-site participation, this abstract book serves as a reference for ongoing education, collaboration, and research development. Readers are encouraged to revisit this collection to explore emerging ideas, identify collaborators, and stay informed on current trends in burn care.

